# Essential Guide of Analysis Methods Applied to Silver Complexes with Antibacterial Quinolones

**DOI:** 10.15171/apb.2018.022

**Published:** 2018-06-19

**Authors:** Aura Rusu, Gabriel Hancu, Silvia Imre

**Affiliations:** ^1^Faculty of Pharmacy, Pharmaceutical Chemistry Department, University of Medicine and Pharmacy of TîrguMureş, Tîrgu Mureș, Romania.; ^2^Faculty of Pharmacy, Department of Analytical Chemistry and Drug Analysis, University of Medicine and Pharmacy of TîrguMureş, Tîrgu Mureș, Romania.

**Keywords:** 4-quinolones, Fluoroquinolones, Analysis methods, Organometallic compounds, Silver

## Abstract

To describe the chemical structure and characterize physico-chemical properties of organometallic complexes it is necessary to use a complex set of analysis methods. Thus, this review has been compiled as a relevant guide which includes the most commonly used methods of analysis in the study of silver complexes with antibacterial quinolones, compounds with promising biological potential. This selection of analysis methods puts on balance the obtained data and the accessibility of the experimental approach. The steps to follow in order to obtain reliable structural information about organometallic complexes of silver, particularly the silver complexes of antibacterial quinolones, are established and presented in the review.

## Introduction

The potential pharmacological effects of the organometallic complexes can be considered appealing and fascinating. An excellent example for this is the antibacterial activity of metal complexes with quinolones.^1,2^ Increased biological effect can be attributed to the interaction of metals with an active organic molecule, and also to the synergism of the antibacterial effect of the complex components.^3,4^ The metal complexes with antibacterial quinolones were summarized in previously published reviews.^5-8^ Moreover, organometallic complexes show other potential pharmacological effects including anti-inflammatory, anti-cancer, anti-oxidant, anti-parasitic, and even insulin-mimetic properties.^3,4,8,9^ Today,the increased bacterial resistance is a worldwide menace; consequently, the discovery of new compounds with an efficient antibacterial effect is a high priority in modern pharmaceutical research. Thereby, organometallic complexes can be a valuable alternative in the fight against bacterial resistance.^10,11^ The study of organometallic complexes also includes the development of efficient methods for their analysis. The current paper presents a review of several analytical methods applied in the analysis of silver complexes with antibacterial quinolones.^12-14^

Analysis methods are used for confirmation of the chemical structure of the obtained organometallic complexes. Thus, the most critical aspect is the determination of sites from quinolone structure where the silver chelation takes place. Quinolone representatives may be coordinated to the silver ion through the 3-carboxyl and 4-pyridone groups.^15,16^ However, most of the quinolones may be coordinated through N-heterocycle from 7 positions.^1,2,17^ Physico-chemical properties, stability and biological effects of the complexes may vary according to coordination site of silver ion.^12^

## Characterization of quinolone-metal complexes

The antibacterial quinolones (4-quinolones) are a class of synthetic antibiotics whose representatives from the first generations have been used primarily for the treatment of urinary tract infections. Newer generations comprise fluorinated derivatives (fluoroquinolones) with broad-spectrum activity. Antibacterial quinolones compounds present a particular mechanism of action by inhibition of DNA replication. Targets for antibacterial quinolones are two essential enzymes involved in DNA replication, DNA gyrase (or topoisomerase II) and topoisomerase IV.^18,19^

**Figure 1:**
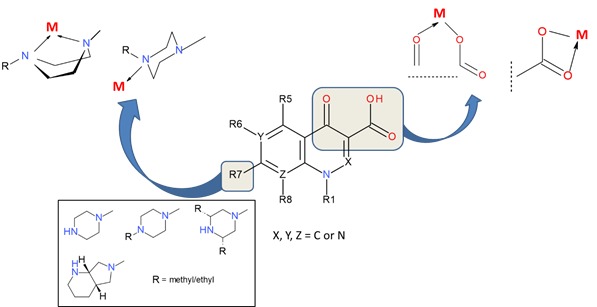
The general structure of antibacterial quinolones and the most common modes of complexation with metal ions (R7 is a heterocycle in various derivatives).

Quinolones are amphoteric compounds with the chemical structure having a carboxyl group at the position 3 and an N-heterocycle (most frequently piperazine) at the position 7. Quinolones interact with metal ions through coordinated interactions that lead to the formation of metal complexes. Consequently, quinolones may participate in the chelation with metal ions as unidentate, bidentate and bridging ligands (Figure 1). A large number of articles focused on metal complexes of antibacterial quinolones and their biological effects have been published and reviewed.^5-8,12^

Among antibacterial quinolones, metal complexes with promising therapeutic effects are the silver complexes.^20-23^Reports regarding several silver complexes have been developed recently to increase antibacterial activity of ligands or acquire new biological effects.^1,2,14,16,24^ The particular coordination of antibacterial quinolones in metal complexes with one silver ion bridge and two silver ions bridge have been previously described.^12^

Obtaining methods of silver complexes with quinolones are relatively simple. Several factors are essential for the future structure of the complex such as silver: ligand ratio, solvents, pH of the reaction mixture, temperature and time. These particular conditions have been described and commented in one of our previous review.^12^

## Analysis methods

The ability of quinolones to coordinate metal ions has been harnessed firstly, in the analyses of pharmaceuticals, biological and food samples. Analysis protocols that have been published comprise different methods such as spectrophotometry,^25-30^ spectrofluorimetry,^31-34^ chemiluminescence,^35,36^ atomic absorption spectrometry,^28^ flow injection and high-performance liquid chromatography.^37-39^

To determine the chemical composition and chemical structure of organometallic complexes, a broad category of methods can be used. After synthesis, elemental analysis (C, H, N) is used to elucidate molecular formula and to describe silver complexes.^40^

The most often applied analysis methods are the spectroscopic ones which provide information regarding the chemical structure and complexation model.

### Flame Atomic Absorption Spectrometry (FAAS)

FAAS was successfully used to quantify the silver content in silver complex with quinolones.^1,14^ This method is applicable when the sample is in solution or readily solubilized; also, the technique is remarkably free from external interferences.^41-44^ However, FAAS is characterized by low sensitivity, is highly expensive and requires extra abilities for the operator.^45^ Consequently, the accessibility of FAAS methods is limited.

### Inductively Coupled Plasma Atomic Emission Spectrometry (ICP-AES)

ICP-AES has been used to analyze a wide variety of sample types.^41^ ICP proved to be a suitable method for the determination of silver percentage in the complexes with quinolones.^24^

ICP-AES replaced to some extent the FAAS technique, but it is also characterized by low sensitivity due to high excitation temperature. Therefore most samples are entirely atomized, and some chemical and spectroscopic interferences may occur. This method requires liquid samples preparation identically to those used for FAAS.^41,46^

### Fourier-transform infrared spectroscopy (FT-IR)

FT-IR is a versatile technique, which can register spectra from samples in liquid, solid or gaseous state.^47^ FT-IR is a useful analytical method for identification and elucidation of chemical interaction in complexes, by comparing the IR spectrum of the complex to ligand IR spectrum. The essential FT-IR band assignments for antibacterial quinolones are characteristic to n(C=O) _carb_ (carboxyl), n(C=O) _pyr_ (pyridone), and n(N^…^H), N atoms from heterocycle (R7).^48^ The recorded FT-IR data disclose that the 4-pyridone and 3-carboxyl groups are implicated in the coordination of silver. Also, registered FT-IR data could be emphasized in studies if the N atoms from heterocycle moiety (R7) are involved in complexation of silver.

The advantages of the FT-IR method are the capacity to record spectra with high signal-to-noise ratios (SNR), possibility to increase the number of scans to improve SNR, and wave number precision. However, the disadvantages of FT-IR technique are well known which are mainly represented by artifacts such as carbon dioxide and water vapor peaks.^49^

### Nuclear Magnetic Resonance Spectroscopy (NMR) 

NMR is the most frequently used analytical method for the elucidation of the chemical structure.^50,51^ In metal complexes of quinolones analysis, ^1^H NMR and ^13^C NMR spectroscopy techniques are used to indicate the chelation of the metal ion to the ligand by the observed chemical shifts in the metal complex spectrum comparative to that of ligand. The significant shifts occur as a result of the implication of some moieties in the coordination process (e.g., nitrogen atoms from piperazinyl substituent).^1,14^

Though, NMR has a lower sensitivity than the MS technique due to amounts of samples of several micrograms. The significant disadvantages of this analytical method are the high cost of deuterated solvents, the price of the spectrometer, and the complexity of the software.^51^

### Mass spectrometry (MS)

MS is an important analytical method used to obtain qualitative and quantitative information about compounds after their conversion to ions.^52^ The different types of ionization sources available today allow characterization of a broad spectrum of compounds, most often based on an electrospray process.^53,54^ However, MS technique is less encountered in the molecular weights determinations of silver complexes of antibacterial quinolones despite the facts that the method has a high sensitivity and requires a short time of analysis.^55^ The coordination of silver ions has lower stability when the silver complexes are analyzed by Electrospray Ionisation Mass Spectrometry (ESI-MS) technique. Another disadvantage is related to their weak solubility in solvents compatible with the MS interface.^14,23^

### Ultraviolet–visible spectroscopy (UV-VIS)

The UV–VIS electronic absorption spectra is a useful and straightforward spectroscopic method for studying organometallic complexes. Mainly, this technique is an easy-to-use, robust, reliable and cheap method which is used to check the identity of drugs. The UV-VIS spectra of parent compounds are compared and evaluated with the UV-VIS spectra of silver complexes.^56^

The electronic absorption spectrum is of two types such as *d-d* spectrum and charge transfer spectrum. The *d-d* spectrum deals with the electronic transitions within the *d-*orbitals. In the charge – transfer spectrum, electronic transitions occur from metal to ligand or vice-versa.^57^

Thus, the electronic spectra of silver complexes measured in the solid state could indicate distinct differences by comparison to the spectra of the parent compound.^14,24,57,58^ For example, the hypsochromic and hyperchromic shifts occur in the main band of levofloxacin spectrum, and a new band occurs in the spectrum of synthesized silver complexes (at 440-450 nm).^14^

### Fluorescence spectroscopy

Antibacterial quinolones present fluorescence properties which are exploited in analytical determination.^59-61^ Fluorescence spectroscopy analysis could be applied for the identification of fluorescent properties of quinolones and their metal complexes and to study the equilibrium constants of complexes.^21,24^

As an example, the ﬂuorescent emission intensity of complex Ag(H-norfloxacin)_2_(NO_3_) in the solid state was larger than that of free H-norfloxacin (about two times). The coordination of norfloxacin to silver ion probably increases the ligand conformational rigidity; thereby this diminishes the nonradiative decay of the (π – π*) excited state of the ligand.^21^ Instead, the ﬂuorescent emission intensity of polymer Ag_4_(Hciprofloxacin)_2_(ciprofloxacin)_2_(NO_3_)]∙4H_2_Odecreases significantly probably due to weak Ag–Ag interactions, leading to a reduced luminescence intensity.^22^ Competitive studies by fluorescence spectroscopy method using 3,8-diamino-5-ethyl-6-phenyl-phenanthridinium bromide (EB) also show the capability of silver complexes to displace EB from the DNA-intercalator EB.^62,63^ Silver complexes can quench the EB–DNA fluorescence. Thus, these experimental data prove that EB was in direct competition with silver complexes at the same binding site for bind to DNA through intercalation.^24,64-66^ Fluorescence spectroscopy is as an adequate, sensitive, reliable and relatively cheap technique and could be useful for analysis of silver complexes and their effects.^67,68^

### Single-crystal X-ray diffraction (XRD)

The most powerful method of analysis of metal complexes is XRD, used to determine the arrangement of atoms of a crystalline solid in three-dimensional space and analysis of the interatomic distances.^69,70^ Meanwhile with the development of the importance o crystallography field the program SHELXL has been improved and became more performant to validate and archive crystal structures. Also, SHELXL is accessible to academics. SHELXTL and SHELXS 97 are improved version of the software.^71^ Both SHELXTL and SHELXS 97 refined by full-matrix least-squares based on *F*^2^(SHELXL 97) ^72^ were used in the analysis of some silver complexes of antibacterial quinolones.^16,17,22,24^

XRD is a fast (under 20 minutes) and powerful technique for identification of unknown compounds which needs minimal sample preparation, wide availability of units, and relatively uncomplicated data interpretation. However, XRD presents some limitation related to the standard reference database, homogeneity of the sample, detection limits, and peak overlay.^73^ Unfortunately, these analysis methods seem to be less accessible to researchers due to the costs of equipment.

### Thermal analysis techniques

The thermal methods complete analysis of silver complexes by revealing their specific thermophysical properties. This category includes Differential Scanning Calorimetry (DSC), Thermogravimetric analysis (TGA), Derivative thermo-gravimetric (DTG) analysis, and Differential thermal analysis (DTA) which gather information about the stability of the complexes (decomposition temperature, kinetics of decomposition, water content).^74^ If the complexation process occurs, the silver complexes will present notable differences of thermal curves from the ligand (melting point, number of decomposition phases, exothermic or endothermic peaks).^1,14,16,23^

DTA and DSC methods offer similar qualitative data. However, DTA can be used at higher temperatures than DSC, but more reliable quantitative information is obtained by DSC.^74^ The precision of these methods is limited due to the dependence of the DTA signals from experimental conditions. DSC is the appropriate thermal method to investigate structure transitions in polymers due to the high sensitivity to any change in the sample or crucible and heating rate. DSC and TGA are both sensitive to the heating rate and sample masses. TGA provides reliability related to mass changes, but due to small amounts of samples, the non-homogeneous materials cannot be tested.^75^

### Density functional theory (DFT)

DFT is an increasingly used computational technique suitable to predict the properties of molecules: molecular structures, atomization energies, vibrational frequencies, electric and magnetic properties, ionization energies, reaction paths etc.^76,77^

The fully optimized geometries of the parent compound and metal complex are presented graphically. Selected bond angles, total energy, charge density, and total dipole moment are also essential data in the characterization of metal complexes. The most common density functionals in DFT model chemistry for some silver complex with levofloxacin, B3LYP (Becke’s three-parameter exchange with Lee, Yang, and Parr correlation functional) for levofloxacin and MWB28 for silver.^14^ The only limitation of the DFT method is the selection of the density functionals. Nowadays, there are no systematic principles of selecting the density functionals. The most published density functionals have been derived by comparison with experimental data.^78^

### Conductivity measurements 

Conductivity measurements provide data about electrolytic or non-electrolytic nature of complexes.^79,80^ Generally, the silver complexes of antibacterial quinolones were soluble in aprotic solvents such as dimethylsulfoxide (DMSO), and dimethylformamide (DMF) and conductivity values are lower than an electrolyte 1:1.,^14,79,80^

Conductivity measurement is an extensive and useful method, with high reliability, sensitivity, and relatively low cost. Also, the measure of conductivity is a rapid and inexpensive technique to determine the ionic strength of a solution. However, it is a non-specific technique due to inability to distinguish between different types of ions.^81^

Based on previously published data we choose to summarise some relevant methods suitable for studies of silver complexes with fluoroquinolones (Table 1).

## Discussion

Correlation and combination of the data obtained from several methods of analysis is a requirement to characterize the chemical structure of a certain organometallic complex. In the preliminary analysis, it is necessary to collect data related to physical properties (appearance, solubility, conductivity, stability) followed by the establishment of the molecular formula that can be determined by elemental analysis combined with FAAS or ICP. To determine the silver content, both FAAS and ICP-AES methods can be used successfully. In a metal complex of an antibacterial quinolone, the polarity of a metal ion is reduced as a consequence to the partial sharing of positive charge with the ligand donor groups and of the overlap with the ligand orbitals. The delocalization of π electrons is increased over the whole chelate ring. Thereby, the lipophilic nature of metal ion is increased. This modification enhances the passage of the complex through the lipid membranes and cells penetration.^4^

The XRD technique is not a very accessible method for researchers, consequently to determine the complexity of the metal ions various combinations of spectroscopic, thermal and computational methods may be used.

Among the spectroscopic methods, FT-IR and MS provide the most unequivocal evidence of silver ion chelation with quinolone ligands. Also, NMR, UV VIS and fluorescence spectroscopy complete and reinforce the data obtained through FT-IR and MS techniques. The DSC seems to be the most appropriate thermal analysis method to asses the behavior of complexes at temperature change in comparison to parent compounds. Nowadays, DTF is a common computational technique suitable to predict the properties of molecules and fulfill the requirements of silver complexes with quinolones characterization.

Obtaining the most accurate chemical structure of silver complexes is closely related to its biological properties, such as the identification of pharmacophores,^82^ interaction with DNA and antibacterial activity,^83,84^ blocking of topoisomerase II enzymes (inhibiting the DNA repair activity) and possible anti-cancer, anti-fungal and anti-parasitic effects.^2,4,18^

**Table 1:** Analysis methods suitable for silver complexes with antibacterial quinolones derivatives.

**Analysis method**	**Purpose**	**Mode**	**Complexes**	**References**
Elemental analysis	Determination of %C, %H şi %N	Comparison found vs. calculated %C, %H şi %N of complexes	Most of the synthesized silver complexes.	
Atomic absorption spectroscopy (AAS)	Determination of % Ag	Comparison found vs. calculated %Ag	Ag(MXF)(H-IMZ)]∙2.5H_2_O	^1^
Flame atomic absorption spectroscopy (FAAS)	Determination of % Ag	Comparison found vs. calculated %Ag	(LVF)_2_Ag(NO_3_) (LVF)_2_Ag(NO_3_)(CH_3_OH) (LVF)Ag(C_6_H_6_O_7_)∙3H_2_O	^14^
Inductively coupled plasma (ICP) spectroscopy	Determination of % Ag	Comparison found vs. calculated %Ag	[Ag_2_(PIP)_2_]_2_·8H_2_O	^24^
FT-IR spectroscopy	Identification and elucidation of chemical structure	Comparison that of ligand IR spectrum, in the range of 400–4000 cm^−1^ by preparing sample pellets with KBr	Most of the synthesized silver complexes.	
^1^H NMR and ^13^C NMR spectrometry	Elucidation of the chemical structure	Indicating the coordination of the fluoroquinolone to the silver ion	Ag(MXF)(H- IMZ)]∙2.5H_2_O (LVF)_2_Ag(NO_3_) (LVF)_2_Ag(NO_3_)(CH_3_OH) (LVF)Ag(C_6_H_6_O_7_)∙3H_2_O	^1,14^
Mass spectrometry	Identification and elucidation of chemical structure	Comparison that of ligand mass spectrum AEI MS 30 mass spectrometer at 70 eV	[Ag_2_(NFX)_2_](NO_3_)_2_ (LVF)_2_Ag(NO_3_) (LVF)_2_Ag(NO_3_)(CH_3_OH) (LVF)Ag(C_6_H_6_O_7_)∙3H_2_O	^14,23^
Electronic spectroscopy	Identification and elucidation of chemical structure	Comparison that of ligand UV-VIS spectrum	[Ag(NLX)_2_] Ag_4_(H-CPF)_2_(CPF)_2_(NO_3_)]∙4H_2_O [Ag_2_(NFX)_2_](NO_3_)_2_ Ag(MXF)(H-IMZ)]∙2.5H_2_O	^1,16,22,23^
Fluorescence spectroscopy	Identification of fluorescent properties of complexes	Comparison that of ligand fluorescence spectrum	Ag_4_(H- CPF)_2_(CPF)_2_(NO_3_)]∙4H_2_O [Ag_2_(PIP)_2_]_2_·8H_2_O	^22,24^
X-ray crystallography/Single-crystal X-ray diffraction (XRD)	Determination of atom arrangements in crystalline solid in three-dimensional space Analysis the interatomic distances	SHELXS 97 refined by full-matrix least-squares based on *F*^2^(SHELXL 97) SHELXTL	Ag_4_(H- CPF)_2_(CPF)_2_(NO_3_)]∙4H_2_O [Ag_2_(H-ENX)_4_](NO_3_)_2_ [Ag(NLX)_2_] [Ag_2_(PIP)_2_]_2_ ·8H_2_O	^16,17,22,24^
DTF calculations	Show full geometry optimizations of the ligand and silver complexes	DFT method in Gaussian09	(LVF)_2_Ag(NO_3_) (LVF)_2_Ag(NO_3_)(CH_3_OH) (LVF)Ag(C_6_H_6_O_7_)∙3H_2_O	^14^
Differential scanning calorimetry (DSC) analysis	Characterisation of the thermophysical properties	DSC curves: (10°C min^-1^temperature increase rate from 40 to 400°C, weight of the samples 3 mg).	(LVF)_2_Ag(NO_3_) (LVF)_2_Ag(NO_3_)(CH_3_OH) (LVF)Ag(C_6_H_6_O_7_)∙3H_2_O	^14^
TGA-DTG-DTA	Gathering information about the stability of the complex (decomposition temperature, kinetics of decomposition, water content)	- TGA: from ambient temperature to 800°C, nitrogen atmosphere - TGA: an oxygen atmosphere (10°C min-^1^temperature increase rate from ambient temperature to 1000°C) - TGA, DTG, and DTA: a dynamic nitrogen atmosphere(20 mL min-^1^), 10°C min-^1^temperature increase rate from 25 up to 800°C	[Ag_2_(NFX)_2_](NO_3_)_2_ Ag(MXF)(H-IMZ)]∙2.5H_2_O [Ag(NLX)_2_]	^1,16,23^
Molar conductivity measurements	Indicates if the compounds are of electrolytic or non-electrolytic nature	10^-3^ M solution of complexes in DMSO	[Ag(PFX)(H-IMZ)]∙2H_2_O (LVF)_2_Ag(NO_3_) (LVF)_2_Ag(NO_3_)(CH_3_OH) (LVF)Ag(C_6_H_6_O_7_)∙3H_2_O	^2,14^
Kinetic data	The activation energy and the size of the ion allow a particular approach of the ligand.	Coats–Redfern and Horowitz–Metzger methods	[Ag_2_(NFX)_2_](NO_3_)_2_	^23^
Measurement of equilibrium constants of complexes	Calculation of the stability constant, K	Fluorescence measurements	Ag(H-NFX)_2_(NO_3_)	^21^

Abbreviations: CPF – ciprofloxacin, ENX – enoxacin, IMZ – imidazole, LVF – levofloxacin, MXF-moxifloxacin, NLX – nalidixic acid, NFX – norfloxacin, PFX – pefloxacin, PIP – pipemidic acid, SHELXS 97, SHELXL 97- software names (programs for the refinement of crystal structures from diffraction data), Gaussian 09 - computational chemistry program, full-matrix least-squares based on F^2^ – refinement method

## Conclusion

The complexation of silver ions with antibacterial quinolones represents a research area of increasing importance, taking into consideration the acute and current problem of bacterial resistance to many marketed drugs. Pharmaceutical properties of antibacterial quinolones can be optimized by obtaining silver complexes with enhanced antibacterial activity or other biological properties. Characterization of metal complexes with derivatives from the quinolone class can be accomplished through a series of analytical methods. The selection of analytical methods is a compromise between the consistency of the corroborated data and the accessibility of the experimental approach. This review describes the main steps to follow for obtaining reliable structural information regarding silver complexes with quinolones and comprises a synthesis of classical and modern analytical methods. The most accurate characterization of an organometallic compound is a prerequisite for determining and understanding the mechanisms of biological effects.

## Ethical Issues

Not applicable.

## Conflict of Interest

The authors declare that they have no conflict of interest.
